# On the origin of rhythmic contractile activity of the esophagus in early achalasia, a clinical case study

**DOI:** 10.3389/fnins.2013.00077

**Published:** 2013-05-21

**Authors:** Ji-Hong Chen, Xuan-Yu Wang, Louis W. C. Liu, Wenzhen Yu, Yuanjie Yu, Liang Zhao, Jan D. Huizinga

**Affiliations:** ^1^Department of Gastroenterology and Hepatology, Renmin Hospital of Wuhan University and Wuhan University Institute of Digestive and Liver diseasesWuhan, China; ^2^Department of Medicine, Farncombe Family Digestive Health Research Institute, McMaster UniversityHamilton, ON, Canada; ^3^Division of Gastroenterology, Department of Medicine, University of TorontoToronto, ON, Canada

**Keywords:** esophagus, achalasia, interstitial cells of Cajal, PDGFRalpha, vagus nerve, enteric nervous system, nitrergic neurons, pacemaker

## Abstract

A patient with early achalasia presented spontaneous strong rhythmic non-propulsive contractions at ~7/min, independent of swallows. Our aim was to evaluate characteristics of the rhythmic contractions, provide data on the structure of pacemaker cells in the esophagus and discuss a potential role for interstitial cells of Cajal (ICC) in the origin of rhythmicity. We hypothesize that intramuscular ICC (ICC-IM) are the primary pacemaker cells. The frequency but not the amplitude of the rhythmic contractions was inhibited by the phosphodiesterase inhibitor drotaverine consistent with cAMP inhibiting pacemaker currents in ICC-IM. The frequency increased by wet swallows but not dry swallows, consistent with stretch causing increase in slow wave frequency in ICC-IM. New studies on archival material showed that ICC-IM were present throughout the human esophageal musculature and were not diminished in early achalasia. Although ICC-IM exhibited a low density, they were connected to PDGFRα-positive fibroblast-like cells with whom they formed a dense gap junction coupled network. Nitrergic innervation of ICC was strongly diminished in early achalasia because of the loss of nitrergic nerves. It therefore appears possibly that ICC-IM function as pacemaker cells in the esophagus and that the network of ICC and PDGFRα-positive cells allows for coupling and propagation of the pacemaker activity. Loss of nitrergic innervation to ICC in achalasia may render them more excitable such that its pacemaker activity is more easily expressed. Loss of propagation in achalasia may be due to loss of contraction-induced aboral nitrergic inhibition.

## Introduction

The objective of this clinical case study was to present a patient with early achalasia that showed strong rhythmic non-propulsive contractile activity in the esophagus that was not dependent on swallows; to show the influence of swallows and the effect of an anti-spasmodic on this rhythmicity; to present new data on the ICC pacemaker network in the human esophagus using archived specimen and to discuss the potential role of ICC in the generation of rhythmic esophageal contractions.

### Case presentation: achalasia patient with spontaneous rhythmic contractions in the esophagus

A 22-year old female (height 158 cm, weight 43 kg, BMI 17.22) presented with a 1.5 year history of dysphagia in the retrosternal area. Initially, her dysphagia could be alleviated within 1–3 min by drinking warm water. Six months before hospital admission (Renmin Hospital of Wuhan University), her dysphagia became more frequent (once every 2–3 weeks), lasting for up to 10 min and could not be relieved by drinking warm water. The retrosternal chest pressure was relieved by regurgitation. These episodes were associated with hypersalivation and profuse sweating. Heartburn and retrosternal chest pain were frequent. Three weeks prior to hospitalization, she was unable to tolerate any solid food orally.

Physical examination showed no focal abnormality. Complete blood count, liver biochemistry, electrolytes and creatinine were normal. An upper gastrointestinal barium swallow study showed typical features of achalasia, demonstrating the bird's beak sign, no barium passed into the stomach during the investigation. Endoscopy, performed after two days of fasting, showed no mucosal abnormality although there was an increased resistance passing the endoscope through the LES.

**Figure 1:**
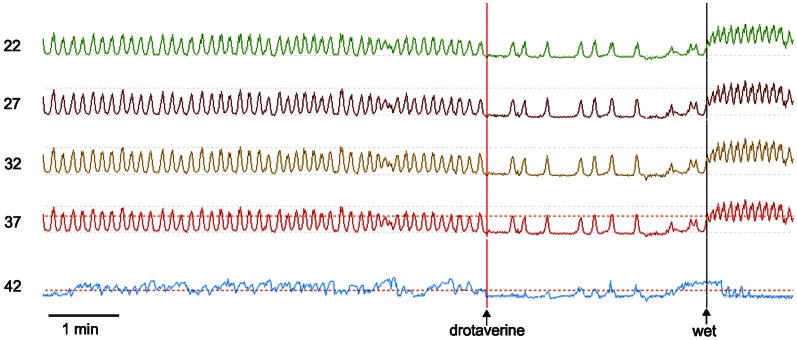
**Manometry tracings of the achalasia patient.** Rhythmic simultaneous contractions occur at 7/min. Drotaverine inhibits the frequency without changing the amplitude. Drotaverine was given about 1 min before the vertical line was placed on the recording. A subsequent wet swallow (wet) markedly increased the frequency. The numbers at the left are the distance of the manometry port in cm away from the nasal edge. The bottom trace is the LES where both drotaverine and a wet swallow are seen to reduce the LES pressure. The amplitude of the spontaneous rhythmic contractions were between 35 and 45 mm Hg. The maximum LES pressure during this recording period was 30 mm Hg.

**Figure 2:**
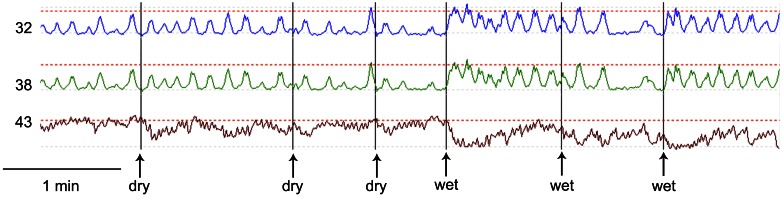
**Comparison of dry swallows (dry) and wet swallows (wet).** The first dry swallow has no effect on amplitude or frequency of the rhythmic contractions. Subsequent dry swallows tend to decrease the amplitude of the contractions. The first and third wet swallows markedly increase the frequency. The amplitude of the spontaneous contractions were between 20 and 40 mm Hg. The maximum LES pressure during this recording period was 50 mm Hg.

Esophageal manometry using a 8-channel water perfused system with a Dent sleeve for LES pressure, showed high LES pressure and regular spontaneous rhythmic non-propagating contractions in the esophageal body at a frequency of 7/min, with an amplitude between 48 and 51 mm Hg (Figures 1, 2). An initial dry swallow did not affect the contractile activity but two subsequent dry swallows decreased amplitude and frequency (Figure 2). Subsequent wet swallows, using 20 ml of warm water, increased the frequency to 8/min and also increased the amplitude (Figure 2). Drotaverine hydrochloride (40 mg intramuscular) was given to reduce the LES pressure; this markedly reduced the contraction frequency from 7 to 3/min without a significant effect on the amplitude; a subsequent wet swallow increased the frequency to 9/min and also increased the contraction amplitude (Figure 1). Significant swallow-induced LES relaxation was still present.

Pneumatic balloon dilatation of the LES was performed uneventfully. The balloon was 35 mm in diameter, 80 mm in length, obtained from EndoFlex Germany. A small amount of blood was seen on the balloon after the dilation procedure. Omeprazole 40 mg iv daily was administered for 3 days. Thereafter the diet was advanced gradually as tolerated. By day 5, liquid meals passed down the stomach without pain. The patient was discharged 7 days after the dilatation. At the 23-month follow up visit, she reported a normal eating habit without any gastrointestinal symptoms.

### Reduction of nitrergic nerves in early achalasia, but no change in ICC

**Figure 3:**
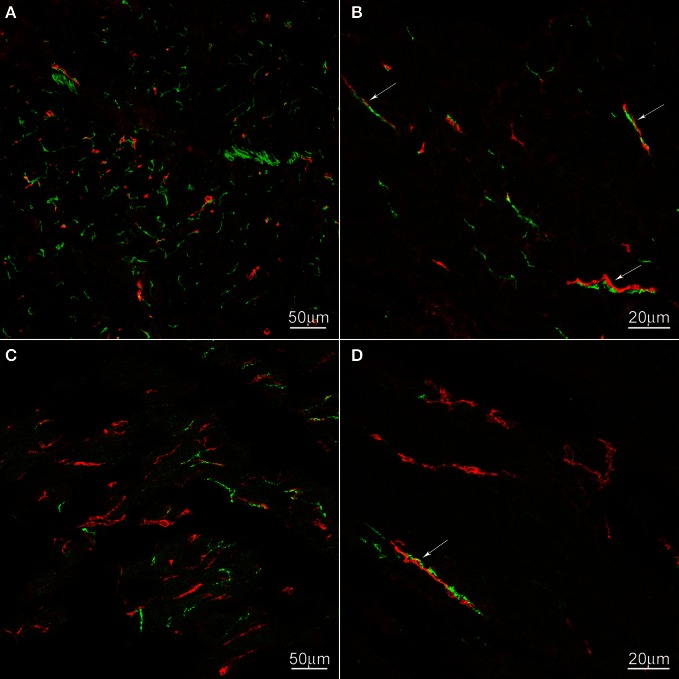
**c-Kit (red) and nNOS (green) immunoreactivities in control (A,B) and achalasia patient (archived specimen) (C,D).** No density difference was not found in ICC between control and the achalasia patient. nNOS nerve density was markedly decreased in the achalasia patient (panel **C,D**). In control esophagus, nNOS positive nerves are frequently and intimately associated with ICC (arrows). These close associations are also occasionally found in the achalasia patient (arrows). In **(A)** and **(B)** the cells are cut cross-sectionally, in **(C)** and **(D)** the cells are cut along their long axis.

We hypothesized that ICC might be responsible for the rhythmicity and sought to find structural evidence for or against this hypothesis. Using archival material, we compared tissues from a patient with achalasia for a period of 2 years (similar to our case study patient) with control tissues. There was no noticeable change in ICC density in the esophageal musculature of the achalasia patient; ICC-IM were distributed within the esophageal muscle coat scattered among the smooth muscle cells and along the connective tissue septa (Figure 3). In contrast, nNOS nerve density was markedly decreased in the achalasia patient (Figure 3) consistent with a previous study (Zarate et al., 2006) where patients with ~1 year achalasia showed a nitrergic nerve density of 0.32% of total area (*n* = 4) compared to 1.86% in control tissue. In fact, two of the patients with 1.5 years duration of symptoms did not have any nNOS positivity (Zarate et al., 2006). Others also found that loss of nitrergic nerves preceded loss of ICC (Watanabe et al., 2002). In control esophagus tissue, nNOS positive nerves were frequently and intimately associated with ICC-IM both within the smooth muscle bundles and within the connective tissue septa (Figure 3). In the achalasia patient tissues, close associations between nNOS nerves and ICC were rare due to reduced presence of nNOS nerves.

### Platelet-derived growth factor receptor alpha (PDGFRα) positive cells are abundant in the human esophageal musculature and form a network with ICC

**Figure 4:**
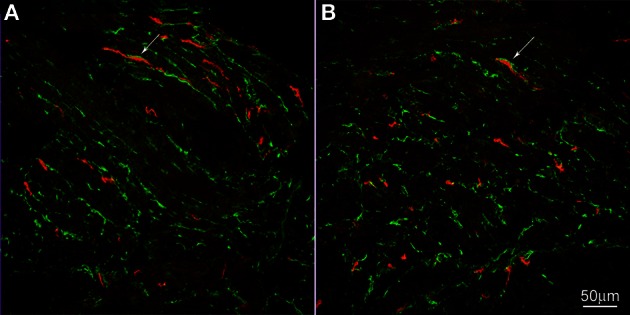
**c-Kit (red) and PDGFRα (green) immunoreactivities in control (A) and achalasia patient (B) (archived specimen).** ICC and PDGFRα positive cells shared a similar distribution in esophagus. They were very close but distinct from each other. Intimate connections were found between them (arrows). No difference was seen in their density and relationship to each other in control patients **(A)** and the achalasia patient **(B)**.

Electron-microscopists have asked our attention for many years to a special type of cell that has many characteristic of a fibroblast, but is not a typical fibroblast and hence was given the name of fibroblast-like cell. Recently, more knowledge about this cell has come forward after the discovery that they contain the platelet-derived growth factor receptor alpha (PDGFRα) (Iino et al., 2009; Chan et al., 2010; Keef and Cobine, 2012). Electron microscopy studies show that, unlike typical fibroblasts, the fibroblast-like cells have gap junction communication with smooth muscle cells shown in mouse small intestine (Horiguchi and Komuro, 2000). In both rat and mouse small intestine, the fibroblast-like cells have many synapse-like close contacts with nerve varicosities (Komuro and Seki, 1995). In the rat colon, the fibroblast-like cells were shown to have gap junction contact with each other and with ICC and close contact with nerves was also confirmed (Wang et al., 2009). In full thickness gastric biopsies of healthy individuals intramuscular PDGFRα were abundant and were shown to contain SK3, a small conductance K channel (Grover et al., 2012). Patients with gastroparesis, which showed loss of ICC, did not show loss of PDGFRα cells (Grover et al., 2012). In the human pylorus and colon, these cells formed apparent networks in both muscle layers, around the myenteric ganglia and at the inner edge of the circular muscle layer (Vanderwinden et al., 2002). Purinergic nerves appear to preferentially innervate PDGFRα cells to cause hyperpolarization, via the SK3 channels; this may contribute to setting the membrane potential of smooth muscle cells and ICC to which they are electrically coupled (Vanderwinden et al., 2002). We show here that these PDGFRα-positive fibroblasts are abundant in the human esophageal musculature and have close connections with ICC-IM of the esophageal circular muscle (Figure 4). No apparent difference was seen in their distribution, quantity or their relationship with ICC in tissue from the achalasia patient compared to controls (Figure 4). Although ICC were loosely dispersed in the musculature, the PDGFRα positive cells were more numerous and together they formed a dense network (Figure 4).

### Details on archival material and immunohistochemistry methods

Control surgical samples were obtained from patients having surgery for carcinoma of the esophagus or stomach without neoplastic infiltration of the cardia, tissue from an achalasia patient with symptoms for 2 years was obtained due to performance of myotomy to alleviate LES pressure; this material was archived after a previous investigation (Zarate et al., 2006). For the present study, ten μ m frozen sections were cut and double stained for either c-Kit/nNOS or c-Kit/PDGFRα. After blocking non-specific antibody binding with 2% bovine serum albumin, sections were incubated with monoclonal mouse anti-c-Kit (Ab 81, 1:100, Santa Cruz Biotech, Santa Cruz, CA, USA) overnight and then with Cy3 donkey anti mouse IgG (1:500) for 1 h. After rinsing with buffer, sections were incubated with either polyclonal rabbit anti-nNOS (1:3000, Chemicon, Temecula, CA, USA) or polyclonal rabbit anti-PDGFRα (R&D Systems, Cedarlane, Burlington, Canada) overnight and then with either Alexa 488 donkey anti rabbit IgG (1:200, for nNOS staining) or Alexa 488 donkey anti-goat IgG (1:200, for PDGFR a staining) for 1 h. All the secondary antibodies were from Jackson Immuno Research (West Grove, PA). 0.05 M phosphate buffered saline (PBS, pH 7.4) with 0.3% Triton X-100 was used for all antibody dilution and rinsing. Controls to assess non-specific staining were carried out by omitting primary antibodies from the incubation solutions. Reactions were examined and pictures were taken with a confocal microscope (Zeiss LSM 510, Germany) with excitation wavelength appropriate for Alex 488 (492 nm) or Cy3 (570 nm).

## Background

### Clinical picture of achalasia

Achalasia is an esophageal disorder characterized by a lack of peristaltic contractions in the smooth-muscle portion of the esophagus and failure or a diminished capacity of the LES to relax (Paterson, 2001; Rohof and Boeckxstaens, 2011). Typical symptoms include progressive solid and liquid dysphagia, regurgitation, chest pain, and weight loss. However, the symptoms and features are heterogeneous and not all “typical” features need to be present (Agrawal et al., 2008; Kushnir et al., 2012; Morera and Nurko, 2012). Esophageal manometry is the gold standard to establish the diagnosis for achalasia. The current therapeutic goal is to relieve the LES pressure to allow the esophageal content to pass into the stomach (Rohof and Boeckxstaens, 2012). Pneumatic dilatation is an effective treatment although patients may require multiple dilations during their lifetime (Richter, 2012). Laparoscopic cardiomyotomy is also frequently employed (Beck and Sharp, 2011). Botulinum Toxin injection to the LES via endoscopy can be used, however, it is less effective and durable than pneumatic dilation. Medical therapies using calcium channel blockers and nitrates are generally ineffective.

The primary cause of the altered motor functions is thought to be a reduction in nitrergic nerves (Paterson, 2001; Rohof and Boeckxstaens, 2011). Numerous studies have shown a reduction in nitrergic nerves of the esophagus of achalasia patients; a condition that progresses over years into loss of ganglia and excitatory neurons (Kraichely and Farrugia, 2006).

### Sphincter relaxation in healthy controls and achalasia

The relaxation of the LES is thought to be primarily but not exclusively due to activity of nitrergic nerves (Kraichely and Farrugia, 2006). This is supported by data from nNOS knockout mice; most swallows in these animals are followed by a poor LES relaxation, although some animals have near normal relaxation (Sivarao et al., 2001). In achalasia, it is logical to assume that loss of nitrergic neurons is responsible for the lack of swallow-induced LES relaxation and high resting LES tone. ICC have been hypothesized to be essential for transmission of nitrergic neural activity to smooth muscle (Ward et al., 1998) and in that way ICC ought to play a role in poor nitrergic innervation; however, in mice without ICC-IM, the LES relaxation was normal (Sivarao et al., 2001). Recent evidence from the mouse fundus shows that a dual pathway exists such that both direct innervation to smooth muscle and indirect nitrergic innervation via activation of guanylate cyclase in ICC occurs in the mouse fundus (Groneberg et al., 2013). ICC is likely not a limiting factor in early achalasia since there is little if any loss of ICC in the first 2 years of achalasia symptoms (Zarate et al., 2006). In advanced achalasia, it will be the loss of nitrergic nerves and not loss of ICC that is the predominant factor in loss of nitrergic inhibition.

### Esophageal body peristalsis in healthy controls and loss of peristalsis in achalasia

Similar to other regions of the gastrointestinal tract, the esophagus has overlapping mechanisms to generate propulsive contractions. Although the vagus is the major driver of swallow-induced peristalsis under normal conditions, the enteric nervous system can provide this function on its own and takes part in normal swallow-induced vagally orchestrated peristalsis; the two systems work in a coordinated fashion (Diamant, 1989). A swallow, or content in the esophagus or esophageal distention activate vagal sensory nerves that provide input to the swallowing center in the brain. This creates primary peristalsis in case of a swallow and secondary peristalsis in case of esophageal content due to activation of vagal and subsequent enteric excitatory nerves dominated by cholinergic but also by peptidergic nerves, whereas the relaxation occurs primarily but not exclusively due to activation of vagal and enteric nitrergic nerves. A liquid bolus may go down the esophagus by gravity followed at the tail end of the bolus by a peristaltic contraction (Goyal and Chaudhury, 2008). The swallowing centre in the brain provides sequential activation of the circular muscle to propel the bolus in the striated muscle. The dominant output is actually inhibition, which is easily demonstrated with repetitive swallows with an interval of 5 s or less, then, no contraction occurs before the end of the last swallow (Biancani et al., 1999). Similarly, when a second swallow is initiated while the contraction associated with the first swallow is still ongoing, this contraction will be inhibited in both the striated and smooth muscle portion of the esophagus. The inhibition after a swallow shows a gradient in duration, creating a latency of the contraction that occurs when the inhibition terminates, the more distal the longer the latency, hence this gradient in latency can account for the apparent propagation of the contraction.

The peristalsis in the smooth muscle part of the esophagus may involve sequential vagal activation (Diamant, 1989) but can also occur by simultaneous activation of the musculature by vagal excitatory nerves, with the latency of contraction provided by vagal and enteric inhibitory neurons (Biancani et al., 1999). Secondary peristalsis is thought to occur through similar mechanisms with content in the esophagus providing the stimulus. In addition to the neurally driven peristalsis, the esophagus has a myogenic (i.e., non-neuronal) control system that can fully orchestrate peristaltic activity including initiation and propagation. It is noteworthy that the velocity of spontaneous and electrically stimulated myogenic peristaltic activity is similar to that of primary and secondary peristalsis in animal models; hence, the myogenic system is intrinsically set to operate in a time frame similar to normal swallow-induced activity (Preiksaitis and Diamant, 1999). The existence of a pacemaker is suggested by the observation that in healthy persons, rhythmic propagating contractions can occur at a frequency of 4–8/min, not initiated by swallowing or intestinal content (Nixon and Koch, 1989). In the normal cat esophagus, propagating contractions can occur spontaneously and can be evoked by distention, they are associated with electrical slow wave activity suggesting association with a myogenic pacemaker since they persist in the presence of the neuronal blocker TTX (Andreollo et al., 1988; Preiksaitis and Diamant, 1999). Also in the opossum, vagally-induced (Rattan et al., 1983) or balloon distention-induced (Paterson, 1989) contractions were associated with electrical slow waves with superimposed action potentials recorded in the musculature. In the presence of TTX, bethanechol can produce rhythmic propulsive contractions in the cat esophagus; also, in the presence of TTX, hence without any neuronal action potential generation, a proximal stimulus can evoke a contraction that propagates distally (Preiksaitis and Diamant, 1999). In the opossum esophagus, direct muscle stimulation caused contractions that propagated in both oral and aboral direction at the same velocity as swallow induced contractions, suggesting a myogenic mechanism of propulsion since neural programs have been shown to exclusively produce distal propulsion (Sarna et al., 1977). Hence, the extrinsic and intrinsic nervous systems and the myogenic control system can fulfill peristaltic activity independently but under normal conditions they will facilitate each other's function. After Sarna et al. suggested that it is the main mechanism of propulsion (Sarna et al., 1977), it is still not known how much the myogenic system is called upon under normal healthy conditions.

In achalasia, immune cells, in particular cytotoxic T lymphocytes, penetrate the myenteric plexus; the first injury observed is the loss of enteric inhibitory nitrergic neurons (Kraichely and Farrugia, 2006). Aperistalsis of the esophageal body is thought to be due to loss of neurons (Kraichely and Farrugia, 2006) such that the aboral relaxation reflex does not materialize. It is also theorized that LES dysfunction may play a role since peristalsis can return when myotomy is performed (Kraichely and Farrugia, 2006). It was speculated that high LES pressure might cause secondary achalasia in patients with anti-reflux surgery (Stylopoulos et al., 2002; Kraichely and Farrugia, 2006). In seven patients who underwent Nissen fundoplication, with an average LES pressure of 16.8 mm Hg, developed secondary achalasia with non-peristaltic esophageal contractions (Stylopoulos et al., 2002). In an animal model of achalasia, a Gore-Tex band, 1 cm wide and 110% of the esophageal circumference in length, was placed around the gastroesophageal junction of opossums to prevent relaxation of the LES during swallowing (Khajanchee et al., 2003). This resulted in the development of vigorous achalasia represented by high-amplitude repetitive simultaneous contractions that was relieved by band removal.

## Discussion

Our study demonstrates spontaneous rhythmic contractions at 7/min in a patient with achalasia symptoms for 1.5 years. Zhang and Diamant observed spontaneous rhythmic contractions in patients with achalasia (Zhang and Diamant, 1994) although this was not observed in another comprehensive study where rhythmic activity was only seen to be swallow-induced (Jee et al., 2009). In recent years, achalasia is classified according to high-resolution manometry features. The patient described in this study would likely be classified as type 2 achalasia according to the Chicago classification (Bredenoord et al., 2012), because of the presence of pan-esophageal pressurization.

### Do the rhythmic contractions in achalasia have a neurogenic or myogenic origin?

A true simultaneous contraction would occur with vagal stimulation without the vagal inhibitory and enteric inhibitory components present. If rhythmic simultaneous contractions were to be exclusively mediated by the vagus, then an extremely stable vagal pacemaker would have to be postulated. Another possibility is that the vagus provides ongoing stimulation and that the musculature responds with rhythmic contractions because of the existence of a myogenic pacemaker that periodically depolarizes the muscle on a continuing basis and forces the stimulus to create rhythmic phasic contraction since only when slow waves are present would the musculature reach sufficient excitation to generate contractions. It is of obvious interest to look for a pacemaker in the vagus, but we already know that a myogenic pacemaker exists. When KCl is given to muscle strips of the opossum esophagus, rhythmic contractions with at a constant frequency develop (Weisbrodt and Christensen, 1972). In the *in vitro* whole cat esophagus, rhythmic circular muscle contractions occurred upon distention by water from a manometry tube, associated with electrical slow wave activity not sensitive to TTX (Andreollo et al., 1988; Preiksaitis and Diamant, 1999). Also in the human esophagus, *in vitro*, TTX insensitive rhythmic activity was associated with electrical slow wave activity (Preiksaitis and Diamant, 1995).

The rhythmic nature of the contractions may have a myogenic origin, but is the stimulus to the musculature to generate contraction myogenic or neurogenic? Contractions in the gastrointestinal tract are often caused by a neural stimulus but myogenic contractions are also common. The typical propulsive contractions of the guinea pig stomach were seen to persist in the presence of nervous blockade (Hirst and Edwards, 2006). Peristaltic contractions in the mouse and rat small intestine are readily observed in the presence of the nerve conduction blocker TTX (Huizinga and Lammers, 2009) In the guinea pig and rat colon, both neurogenic and myogenic motor patterns can be identified (D'Antona et al., 2001; Chen et al., 2013) with distention being the major stimulus of myogenic contractions.

The forceful nature of the rhythmic contractile activity observed in patients in the early stage of achalasia is likely in part caused by reduction in nitrergic nerves, which leaves the myogenic and neural excitation unopposed by the normally significant inhibition by nitric oxide (Anand and Paterson, 1994). Behar and Biancani, focused a study on the pathogenesis of simultaneous contractions in functional dyspepsia (Behar and Biancani, 1993). In this study, and also in many other studies, contractions were actually not simultaneous but had an increased velocity with a delay changing from ~6 s to ~3 s. This change in delay was hypothesized to be due to changes in inhibitory neural activity. Their conclusion was that “simultaneous” swallow induced contractions are due to ineffective neural inhibition and that spontaneous “simultaneous” contractions are due to abnormal release of acetylcholine. Interestingly, in another case of rhythmic contractions in a patient with more advanced achalasia at Renmin Hospital, atropine did not influence the force or frequency of contractions.

The spontaneous rhythmic contractile activity in early achalasia likely has a myogenic pacemaker underlying it; the generation of the forceful contractions is likely due to loss of nitrergic inhibition of both neural and myogenic stimuli for contraction. If loss of nitrergic innervation is the only significant feature of early achalasia, then clearly a pure myogenic control system is not capable of fulfilling *propulsive* contractions.

### Are ICC responsible for the rhythmicity of contractions in early achalasia?

Rhythmicity in the gastrointestinal musculature is most often provided by networks of ICC, most prominently by the interstitial cell network that is associated with the myenteric plexus (the ICC-MP) and by ICC that form a network within the muscle layers, the intramuscular ICC (ICC-IM) (Huizinga et al., 1995; Der-Silaphet et al., 1998; Thomsen et al., 1998; Timmermans, 2001; Hirst and Edwards, 2006; Sanders et al., 2006; Huizinga and Lammers, 2009). ICC-MP in the esophagus are rare such that they do not form a network necessary for pacemaking. ICC are also found dispersed within the esophageal muscle layers, the intramuscular ICC (ICC-IM). The ICC-IM are more numerous but also do not form a dense network in the smooth muscle portion of the esophagus, suggesting that they may not by themselves create a pacemaker network. However, we show here that fibroblast-like or PDGFRα-positive cells are connected to ICC-IM in the esophagus and together with ICC-IM form a dense network that may provide pacemaking and a pathway for coupling and propagation of the electrical slow waves.

The role of ICC in esophageal rhythmic contractions has received little attention. This is likely because ICC in the myenteric plexus are rare and intramuscular ICC are sparse especially in the upper esophagus and do not form a network similar to their counterparts in the stomach (Streutker et al., 2007; Huizinga et al., 2008). Vagal nerves may also provide rhythmicity. In rats, rhythmic contractions at ~38/min were induced by fixed balloon distention and were shown to be correlated with rhythmic discharges from the nucleus ambiguous innervating the striated muscle (Lu and Bieger, 1998). The rhythmic contractions in the feline esophagus associated with myogenic slow wave activity were around 1–8/min (Preiksaitis and Diamant, 1999); the observed rhythmic contractions in the present study fell into this range.

The present study shows properties of the esophageal rhythmicity that are consistent with ICC-IM being the pacemaker cells. Drotaverine reduced the frequency of phasic contractions without affecting the amplitude (Figure 1) suggesting that drotaverine specifically inhibited the pacemaker frequency. Drotaverine is a papaverine-like antispasmodic acting through inhibition of phosphodiesterase (Muravyov et al., 2007). This suggests that the pacemaker mechanism is affected by changes in intracellular cAMP. Interestingly, cAMP has been shown to reduce the frequency of pacemaker currents and slow waves in murine gastric ICC (Kim et al., 2002). A wet swallow immediately increased the contraction frequency (Figure 1) suggesting that the pacemaker was also sensitive to stretch or distention. Indeed, ICC-IM have been shown to be stretch sensitive in the murine stomach (Won et al., 2005); stretch caused a marked increase in slow wave frequency that was dependent on the presence of ICC-IM. The suggestion that the esophageal pacemaker is sensitive to stretch is consistent with a study that shows that the frequency of balloon-induced repetitive contractions increases with increasing balloon distention in healthy volunteers (Andreollo et al., 1988). In the rat fundus (Powley et al., 2008) as well as in the cat esophagus (Huizinga et al., 2008), ICC-IM have been shown to be part of the vagal afferent pathway and were suggested to be a critical part of the vagal mechanoreceptors, the intramuscular arrays (IMA), indicating that direct communication between vagal nerves and ICC is possible. Shafik recorded a stable rhythmic electrical activity in the esophagus of volunteers using mucosal suction electrodes, at a frequency of 4–7 min and a propagation velocity of 3–6 cm/s (Shafik et al., 2004). The frequency increased upon esophageal balloon distention to 9/min (Shafik et al., 2005). The observation of spontaneous electrical slow waves is consistent with the observation of spontaneous rhythmic peristaltic contractions in routine manometry at the same frequency (Nixon and Koch, 1989).

Rhythmic contractions also occur in the striated portion of the esophagus. Jee et al suggested that they originated in the upper esophageal sphincter (UES) since there they had the highest amplitude (Jee et al., 2009). Zhang and Diamant noted that rhythmic esophageal contractions were not common when the UES did not show them (Zhang and Diamant, 1994). This does not exclude ICC from playing a role in the origin of the rhythmicity since ICC occur in the human striated muscle of the esophagus (Faussone-Pellegrini and Cortesini, 1986) as well as in the mouse (Rumessen et al., 2001) and pig (Wu et al., 2003). Furthermore, when cell clusters were isolated from the rat esophagus, rhythmic contractions developed, dependent on the presence of ICC (Ludlow et al., 2013).

The above-described observations show that the esophagus has a myogenic control system that can fully orchestrate peristaltic activity. The ICC are the likely candidate for pacemakers and the network of ICC and PDGFRα positive fibroblast-like cells may provide a pathway for pacemaker (slow wave) propagation. Rhythmic contractions are a feature of early achalasia and are not observed in later years, this is consistent with a role for ICC since significant loss of ICC likely only starts 2 years after the onset of achalasia (Zarate et al., 2006).

### Is ICC pacemaking inhibited in healthy control subjects?

ICC-IM in the esophagus are intimately connected to intrinsic nitrergic nerves as shown in Figure 3 and in previous studies on the human esophagus (Zarate et al., 2006) and murine LES (Ward et al., 1998). In addition, the ICC in the striated muscle of the mouse appear to be uniquely associated with intrinsic inhibitory nerves, primarily nitrergic nerves (Rumessen et al., 2001; Wu et al., 2003). Paterson showed that balloon distention in the opossum esophagus generates myogenic contractions that increase in amplitude after TTX administration, indicating physiologically that the musculature is under tonic inhibitory control (Muinuddin and Paterson, 2001). In achalasia, the connection between ICC and nitrergic nerves is severely diminished because of loss of the nerves (Zarate et al., 2006), hence it is logical to suggest that in achalasia ICC have lost an inhibitory factor, which would make their pacemaker activity easier to evoke or be expressed.

### Why do the rhythmic contractions in early achalasia not propagate?

Nitrergic nerves are an important factor in propagation of primary and secondary peristaltic contractions in the esophagus. Nitrergic nerves are the primary determinants of deglutition-induced inhibition determining the gradient in latency of the propulsive contraction along the esophagus. Indeed, inhibition of nitrergic innervation increases the propagation velocity (Anand and Paterson, 1994) hence loss of nitrergic innervation is a logical explanation for the simultaneous nature of swallow induced rhythmic contractions in the achalasia patient (Behar and Biancani, 1993). Loss of nitrergic nerves is however, not essential for simultaneous contractions. They occur occasionally in healthy persons and are more numerous in patients with spasmic esophageal contractions. In these patients simultaneous contractions can alternate with normal propulsion. Transient presynaptic inhibition of nitrergic nerves could be responsible for this, although this has never been demonstrated. Activity of nitrergic nerves can be blocked by presynaptic action (Esplugues, 2002); this has been demonstrated to occur for example via alpha-adrenoceptors in the canine ileocolonic junction (Boeckxstaens et al., 1993), via muscarinic receptors on cerebral perivascular nitrergic nerves (Liu et al., 2002) and varicosities of serotonergic interneurons have been shown to appose nitrergic neurons in the guinea pig small intestine (Neal and Bornstein, 2007).

The spontaneous *rhythmic* contractions occurring in the achalasia patient described here are not generated by classical swallow or bolus-induced reflexes using aborally projecting nitrergic nerves. It is possible that spontaneous contractions themselves via sensory neurons (Kunze et al., 1999) activate aborally projecting nitrergic nerves that might be essential for their downward propulsion. Paterson found that the descending inhibition caused by balloon distention of the esophagus and the contraction occurring upon balloon deflation is actually initiated by a *contraction* evoked by the balloon-induced stretch (Muinuddin and Paterson, 2001); this reflex was abolished by blockade of nitric oxide synthesis (Paterson and Indrakrishnan, 1995). Hence the loss of nitrergic nerves in achalasia might render the spontaneous rhythmic contractions stationary. However, myogenic mechanisms might be responsible for the simultaneous nature of the contractions. In the intestine, propagation of the contractions that find their rhythm determined by the slow waves generated by ICC-MP follow the propagation of slow waves (Lammers and Stephen, 2008) that occurs though the network of ICC in anal direction. Is this the case in the esophagus with spontaneous rhythmic contractile activity? Rhythmic contractile activity is associated with slow wave activity, shown in the cat esophagus (Preiksaitis and Diamant, 1999) and the human esophagus (Preiksaitis and Diamant, 1995). The functional existence of a pathway for myogenic propagation of slow waves was proven by earlier experiments (Sarna et al., 1977; Preiksaitis and Diamant, 1999) and here we provide structural data to show the existence of a network of ICC and PDGFRα cells and we speculate that this network is the likely candidate for the conduit of electrical coupling and the pathway of slow wave propagation in the esophagus. Two factors have a strong influence on myogenic propagation velocity, the rate of rise of the slow wave (which determines how quickly the next cell is depolarized) and the strength of cell-to-cell coupling provided by gap junctions. The gap junctional conductance is likely under neural control, possibly by nitrergic nerves, similar to dopaminergic regulation of gap junctional conductance in retinal cells (Lasater, 1987; Mills et al., 2007) acting through cAMP to open or close gap junctions dependent on the cell type. Nitric oxide reduces cell-to-cell coupling by increasing cGMP in horizontal cells thereby playing an important role in retinal light adaptation (Xia and Mills, 2004). One can speculate that if nitric oxide keeps the ICC gap junctions relatively closed (the propagation of contractions in the esophagus is slow, compare 2 cm/s with 50 cm/s of the cardiac contraction), loss of nitrergic innervation could increase contraction velocity such that it appeared virtually simultaneous. The neural regulation may be affected by achalasia, since nitrergic control of ICC is reduced because of loss of nitrergic nerves.

### Is ICC-related sensory perception in the esophagus abnormal in achalasia?

Lack of normal reflexes in early achalasia is likely caused by absence of nitrergic innervation caused by loss of nerves. But in some patients peristalsis returns after dilatation suggesting that in such cases there was no permanent damage to the enteric nervous system and/or the myogenic pacemaker system. This suggests that other reversible factors may play a role in loss of peristaltic activity in early achalasia. In some patients, failure of the LES to relax causes distention of the esophagus by stalled food, or decreased nitrergic innervation may increase muscle tone (Zhang and Diamant, 1994). So a constant stimulus may be present which is relieved by dilatation. The constant stimulus may desensitize sensory nerves or the vagal mechanosensors: the intramuscular arrays (IMAs) that are coupled to ICC (Powley et al., 2008), which may be in part responsible for lack of primary and secondary peristalsis. Rumessen et al. also postulated that ICC are involved in the sensory information processing in their study on ICC in the striated muscle of the esophagus; they concluded that discrete clusters of ICC in the striated esophagus represent specialized spindle proprioceptors (Rumessen et al., 2001).

## Concluding remarks

We hypothesize that the esophageal ICC are pacemaker cells providing the rhythm to the spontaneous or swallow-induced rhythmic contractions of the esophagus in early achalasia. ICC in the striated and smooth muscle part of the esophagus are extensively innervated by nitrergic neurons. We hypothesize that the pacemaking is normally suppressed by nitrergic innervation that is diminished in achalasia. Rhythmic esophageal contractions are associated with slow waves, and we speculate that they have their pathway for coupling and synchronization of pacemaking as well as propagation provided by the network of ICC and PDGFRα-positive fibroblast-like cells. The lack of propulsion in achalasia maybe due to lack of contraction-induced aboral nitrergic relaxation.

### Conflict of interest statement

The authors declare that the research was conducted in the absence of any commercial or financial relationships that could be construed as a potential conflict of interest.
